# Commentary: Why Study the History of Neuroscience?

**DOI:** 10.3389/fnbeh.2020.00127

**Published:** 2020-08-13

**Authors:** Jeremy Trevelyan Burman, Brianne M. Collins

**Affiliations:** ^1^Theory & History of Psychology Department, Heymans Institute for Psychological Research, University of Groningen, Groningen, Netherlands; ^2^Psychology Department, Providence University College, Otterburne, MB, Canada

**Keywords:** history of neuroscience, history of psychology, historiography, history of science, science education, Thomas Kuhn

Memory is not synonymous with History. Granted, a discipline that doesn't remember its past suffers from a kind of epistemic Alzheimer's—as Brown (2019) put it memorably. This is recognizably pathological, and therefore affords an apparently-compelling answer to the titular question. However, the evocation is limited. Not only is memory not always an accurate impression of the past, but the metaphor also does little here to explain the value of history for science. Nor is it consistent with how specialists in the field, or allied areas, view their subject.

Nearly 60 years ago, Kuhn ([1962] 2012) popularized what is now the mainstream approach to the history of science: the historian's goal is to understand “the historical integrity of that science in its own time” (p. 3). History is therefore no longer memorial, or celebratory, but investigative: *How did what past scientists do make sense to them at the time? How were ideas and discoveries the products of pressures—conditions of possibility, power, governmentality, thinkability—which existed in their contexts?* (see also Burman, 2020).

As a result of adopting this perspective, Kuhn ([1962] 2012) criticized the treatment of history as the writing of tourist brochures. Such memorabilia were then discarded by specialist historians of science, including by historians of the behavioral sciences (broadly conceived; see e.g., Stocking, 1965; Young, 1966). Thus, regrettably, Brown advocates for a return to an approach that has been out-of-date for more than half-a-century.

That said, however, the espoused view is not representative of the field. For instance, Gavrus and Casper (2017) positioned their *History of the Brain and Mind Science* in explicit contrast to that old-fashioned approach. Their perspective is then consistent with scholarship in allied areas (see Furumoto, 1989; Hilgard et al., 1991; Capshew, 2014). Indeed, recent research assumes these historiographical virtues—such as a critical approach, and a focus on unheard voices (or silenced subjects)—then proceeds to derive new insights along lines afforded by several broad themes (Burman, 2018).

Most problematic in the original essay, though, is that Brown failed to follow his own memorial through-line. The metaphor could easily have been made consistent with contemporary historiographical concerns. To wit: *What, or who, have we forgotten and why?* (see Draaisma, [1995] 2000). Indeed, this is what makes historical research so exciting (e.g., Burman, 2015, 2019; Rutherford, 2015). And it's why history is valuable pedagogically: it requires reflection, and perspective-taking, as a function of method.

We want students to be more than tourists who visit Disney's EPCOT resort, then leave thinking they've had an *authentic* cultural experience. We also don't want them to lament the ignorance of those who did otherwise. Instead, we want them to be more humble; to prefer to go to the source, whenever possible, and learn to see things according to how and why those things made sense to the people who held other beliefs. In other words, we want them to learn how to think “from below” (Thompson, 1966; cf. Porter, 1985; Spivak, 1988). To hear those who can't be heard (e.g., Jacyna and Casper, 2012).

Brown's writing, though, is memorable. We thus conclude with something equally so: *History is not about us—it's about them. The goal is not to judge, but to understand. And that's a valuable thing for everyone to learn*. Whether or not you want to work as an Historian after, perspective-taking and deep understanding from within (“cultural competence”) are useful and indeed marketable skills.

## Author's Note

This commentary was prompted originally by a discussion in the Theory and History of Psychology expert-group at the University of Groningen, where related themes are taught in the graduate programme (https://www.rug.nl/masters/theory-and-history-of-psychology/). The draft then developed in conversation between the authors, back and forth, across several iterations. The result was a much longer text, which—following the required word limit—was pruned back and focused on the key issues.

## Author Contributions

JB served as primary author, responsible for broad themes in the historiography of science as they pertain to the history of the behavioral sciences (broadly conceived). BC provided expertise about the history of neuroscience, neurology, and neurosurgery. All authors listed have made a substantial, direct and intellectual contribution to the work, and approved it for publication.

## Conflict of Interest

The authors declare that the research was conducted in the absence of any commercial or financial relationships that could be construed as a potential conflict of interest.
